# Supplementary figures and images for: A bigenic mouse model of FSGS reveals perturbed pathways in podocytes, mesangial cells and endothelial cells
Source: PLoS One. 2019 Aug 28;14(8):e0216261. doi: 10.1371/journal.pone.0216261 (PMC6713350; doi:10.1371/journal.pone.0216261)

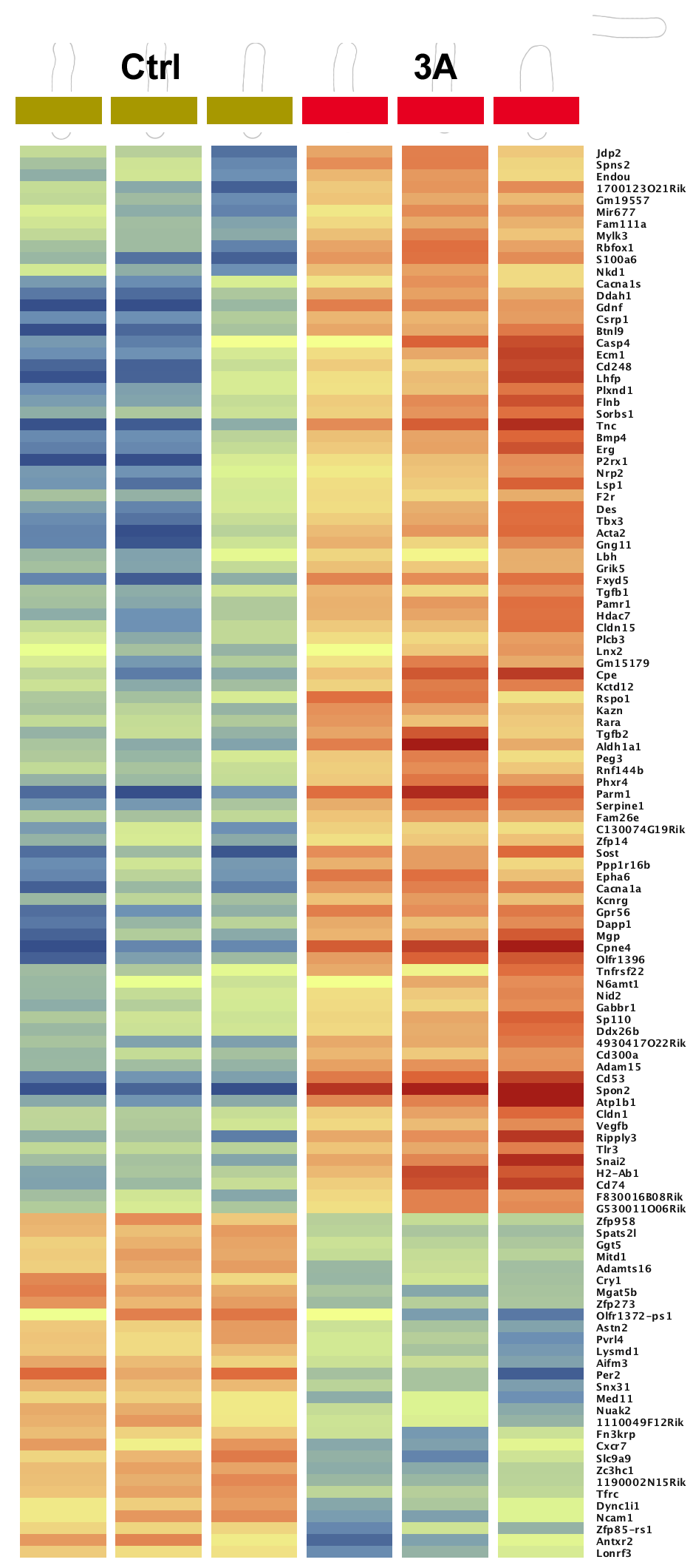

Supplement: S1 Fig — Red indicates high expression and blue corresponds to low expression levels. (TIF) [file pone.0216261.s004.tif]
